# A functionalized self-assembling peptide containing E7 and YIGSR sequences enhances neuronal differentiation of spermatogonial stem cells on aligned PCL fibers for spinal cord injury repair

**DOI:** 10.7150/thno.78448

**Published:** 2022-10-31

**Authors:** Zhiyuan Wang, Shuaijun Jia, Hailiang Xu, Xiaohui Wang, Botao Lu, Weidong Wu, Dageng Huang, Lingbo Kong, Xin Kang, Fang Tian, Lei Zhu, Dingjun Hao

**Affiliations:** 1 Department of Spine Surgery, Honghui Hospital, Xi'an Jiaotong University, Xi'an, China.; 2 Shaanxi Key Laboratory of Spine Bionic Treatment, Xi'an, Shaanxi, China.; 3 Department of Sports Medicine, Honghui Hospital, Xi'an Jiaotong University, Xi'an, China.

**Keywords:** Spermatogonial stem cell, Neuronal differentiation, Self-assembled peptide, Spinal cord injury

## Abstract

**Background:** Spinal cord injury (SCI) induces neuronal death and disrupts the nerve fiber bundles, which leads to partial or complete sensorimotor function loss of the limbs. Transplantation of exogenous neurons derived from stem cells to the lesion site becomes a new neurorestorative strategy for SCI treatment. Spermatogonial stem cells (SSCs) can attain pluripotency features by converting to embryonic stem-like cells *in vitro*. However, differentiating SSCs into lineage-specific neurons is quite difficult and low efficiency.

**Methods:** Immunofluorescence, immunohistochemistry, Western blotting, whole-cell patch clamp, and behavioral tests were performed to verify that self-assembled hydrogels could improve the directional differentiation efficiency of SSCs and the feasibility of SSC-derived neurons in the treatment of spinal cord injury.

**Results:** We developed a novel self-assembled peptide Nap-FFGEPLQLKMCDPGYIGSR (Nap-E7-YIGSR) coated with aligned electrospun PCL fibers to enhance neuronal differentiation of SSCs. The Nap-E7-YIGSR peptide could evenly self-assemble on the surface of PCL fibers, enhanced the materials's hydrophilicity, and improved the SSC affinity of PCL fibers through the stem cell adhesion peptide sequence EPLQLKM domain. In addition, Nap-E7-YIGSR could effectively induce SSC neuron differentiation by activating the integrin β1/GSK3β/β-catenin signaling pathway. Moreover, implanting the induced neurons derived from SSCs into SCI lesion sites in rats resulted in the formation of new relay circuits, myelination, and synapse formation. Furthermore, SSC-derived neurons could survive and function in the spinal cord injury microenvironment, boosting the recovery of locomotion.

**Conclusion:** The combination of the multifunctional peptide and aligned fibers can potentially trigger SSC differentiation to neurons, facilitating neuronal replacement therapy and promoting functional recovery after SCI.

## Introduction

Spinal cord injuries (SCI) are severe damage to the spine, which are frequently caused by a motor vehicle collision and external trauma. Spinal cord injuries inevitably produce partial or complete sensory and motor function loss below the injured area and result in dysfunction of the respiratory, urinary, and gastrointestinal tracts and even lead to multiple organ failure 1, 2. Several factors, such as neuron loss, the limited regenerative capacity of the central nervous system (CNS), disruption of the synaptic connections, and a complex microenvironment, can result in severe and irreversible abnormalities in motor and sensory function below the lesion regions 3, 4. Although internal spinal stabilization, surgical decompression, blood pressure control, corticosteroid shocks, and neurotrophic medicines are normal clinical methods for treating patients with spinal cord injuries, almost none of these improve the patients' motor function appreciably 3. After spinal cord damage, none of these clinical treatments have been able to restore the neuronal loss. Exogenous neuronal replacement therapies are therefore widely investigated in animal models. Several animal studies have shown that neuron and neural stem cell transplantation strategies can improve functional recovery following different types of spinal cord injuries, providing new approaches for the successful treatment of human spinal cord injury in the future and giving hope to patients with spinal cord injury 5-8.

Neural stem cells exist in the healthy spinal cord and are capable of converting into neurons and glia 9, 10. The differentiation and migratory ability of the spinal cord endogenous neural stem cells are activated after spinal cord injury 11. However, the limited number of endogenous neural stem cells in the central canal cannot efficiently and functionally replace necrotic or apoptotic neurons after spinal cord injury 12. For this reason, exogenous neurons and neurons derived from exogenous stem cells represent a viable alternative. To date, several types of stem cells, including embryonic stem cells (ESC), induced pluripotent stem cells, and adult stem cells, can differentiate into various neural lineages 13-16. There are several social and ethical issues linked to embryonic stem cells have a significant impact on their therapeutic uses. Besides, the tumorigenicity of induced pluripotent stem cells also limits their utility for treating spinal cord injury 17.

As a kind of adult stem cells, bone marrow mesenchymal stem cells (BMSCs) are abundantly available and easily accessible without any ethical concerns and tumorigenic risks proven to differentiate into neurons in many studies. However, the proliferation and neuronal differentiation capabilities of BMSCs undergo obvious functional decline with systemic aging 18. Also, this age-related decrease in function is present in other adult stem cells 19, spermatogonial stem cells (SSCs) have the amazing capacity to renew and replicate over the whole lifespan in men, and this ability stays the same regardless of age 20. In addition, previous studies have also confirmed that SSCs can differentiate into multi-lineage cells of the body and are capable of differentiating into a variety of functional neurons 21, 22. Besides, in contrast to other adult-type stem cells, SSCs demonstrate various unique qualities like lack of ethical problems, decreased tumorigenicity, wide availability, a lower host immunological response, no risk of virus contamination 23. Therefore, the differentiated neurons derived from SSCs as the transplanted cells to treat SCI are more suitable for clinical application than other stem cells. However, it is extremely difficult to differentiate SSCs into lineage-specific neurons and the efficiency is low. Therefore, improving their neuronal differentiation efficiency is a technical problem to be solved urgently.

Topographical and chemical cues are two crucial factors for directing the differentiation of stem cells 24-27. Due to their weak adhesion properties, SSCs cannot adhere well to the culture flask. On the contrary, neurons exhibit significant adhesive characteristics, and substrates are essential for neurite contact guidance 28, 29. To aid in the process of neuronal differentiation, it is important to increase the adherence of spermatogonial stem cells *in vitro*. Previous studies have reported that a specific peptide (EPLQLKM, E7) can effectively increase the adhesive efficiency of mesenchymal stem cells (MSC) 30. To our surprise, we found that E7 could also improve the adhesion properties of SSCs. In addition, the YIGSR pentapeptide derives from laminin, which has been shown to promote axonal elongation and motility of central and peripheral neurons 31. YIGSR also facilitates and directs embryonic stem cells to differentiate into neurons 32. However, whether YIGSR has a function in SSC differentiation into neurons is primarily unknown.

To modify the E7 peptide and YIGSR on the surface of the growth substrate, we designed a new self-assembled peptide Nap-FFGEPLQLKMCDPGYIGSR (Nap-E7-YIGSR). This self-assembled peptide contains hydrophobic naphthalene groups originating from 2-naphthaleneacetic acid, and the Nap-FFG terminal has an excellent ability to self-assemble on the hydrophobic surfaces. In addition, the functional groups of E7 and YIGSR are linked by CDPG. As a biologically inert material, the surface of the aligned fibers of Poly (ε-caprolactone) (PCL) has a strong hydrophobicity. Neurons have been shown to develop along aligned fibers 33. However, it is unclear whether the aligned PCL fibers coated with Nap-E7-YIGSR functional self-assembling peptide can improve SSCs adhesion, induce morphological differentiation, and SSCs differentiation to neurons, and increase nerve growth. In addition, since neuronal loss is one of the most significant obstacles to functional recovery after spinal cord injury, transplantation of SSCs-induced differentiated neurons has the potential to restore motor and sensory functions after spinal cord injury. Therefore, we hypothesized that SSCs-derived neurons may integrate into the lesion site and potentially improve neurological function recovery.

In this study, a functional self-assembled peptide Nap-E7-YIGSR was designed and synthesized to coat on the surface of the electrospun PCL membrane with aligned fibers. This functional peptide-coated PCL membrane was seeded with SSCs for neuron differentiation. Immunofluorescence, scanning electron microscopy (SEM), and Western blotting were used to evaluate the efficiency and molecular mechanism of Nap-E7-YIGSR in boosting the differentiation of SSCs to neurons. Finally, we implanted SSC-derived neurons into the SCI rat model to investigate their survival and effectiveness in rebuilding neural circuits. Rat motor function was assessed through histological and electrophysiological tests and gait analysis.

## Materials and Methods

### Ethics statement

All animal experiments were conducted in accordance with Xi'an JiaoTong University guidelines for animal research and use.

### Synthesis of Nap-E7-YIGSR self-assembled peptide

The Nap-E7-YIGSR self-assembling peptide was synthesized by the solid-phase peptide synthesis method, as described previously 34. Briefly, the resin was swelled with N, N-dimethylformamide (DMF), and N-Fmoc protected amino acids were introduced. Then DMF containing 20% piperidine was used as a deprotection reagent, and the coupling reagent consisted of 4-methyl morpholine (NMM) and O-benzotriazole-N, N, N, N-tetramethyl-uroniumhexafluoro-phosphate (HBTU). After repeated cycles of N-terminal deprotection and coupling reactions, the peptide was cleaved from the resin with TFA cleavage reagent and purified by liquid chromatography. The Nap-FFGEPLQLKMC (Nap-E7) peptide was also synthesized for follow-up experiments.

### Fabrication of aligned PCL fibrous membranes

The aligned PCL fibrous membranes were fabricated according to the previously described procedures 35. In brief, 1.5 g of PCL particles (Mn=80000) were dissolved in 10 mL of a chloroform/methanol mixed solution (5:1 v/v). The 15% PCL solution was loaded in a 10 mL syringe with a 21-gauge stainless steel needle and flowed at the rate of 5 mL/h by a micro syringe pump. The distance between the syringe needle and the roller receiver was about 15cm, and the voltage was 16 kV. To obtain the aligned fibers, the roller rotation speed was set to 4000 rpm. The morphology of PCL membranes was observed by scanning electron microscope (SEM; S-3400N; HITACHI, Tokyo, Japan) at an accelerating voltage of 5 kV.

### Surface modification and hydrophilicity

The aligned PCL fibrous membranes were immersed in a 4 mL solution containing Nap-E7 (0.5 μmol/mL) or Nap-E7-YIGSR (0.5 μmol/mL) at 37 °C for 24 h. In the control group, the aligned PCL fibrous membranes were immersed in phosphate-buffered saline (PBS) for 24 h at 37 °C. After removing the peptide solution, membranes were rinsed three times with PBS. In order to observe the binding and distribution of self-assembled peptides on the surface of the fibers, Nap-E7 was labeled with FITC, and Nap-E7-YIGSR was labeled with rhodamine. The peptide distribution and binding were observed under a fluorescence microscope. The hydrophilicity of PCL membranes in each group was evaluated by the contact angle measured by a contact angle goniometer.

Coating peptides amounts were measured following the procedures. In brief, we submerged 1 cm^2^ electrospun PCL membrane in 4 mL of NAP-E7, NAP-E7-YIGSR solution with a concentration of 0.5 μmol/mL for 24 h. After 24 h, we rinsed the modified PCL membrane with pure water for 3 times, 1 mL each time, and collected it, and mixed it with the original solution to obtain 7 mL of NAP-E7, NAP-E7-YIGSR solution. The total amount of peptide in both solutions was measured by BCA Protein assay.

### Isolation, purification, and identification of primary SSCs

SSCs were isolated and purified from SD rats, as reported previously 20. In brief, seminiferous tubules were isolated from testis of 23-25 days old rats and digested in a solution containing collagenase type I (1 mg/mL), trypsin (2.5 mg/mL), and DNase I (200 μg/mL) at 37 °C for 15 min. The cells were dispersed by repeated pipetting and fetal bovine serum (FBS) was added to stop digestion. After filtered through 300 μm filters and centrifuged at 2000 rpm for 5 min. Primary SSCs were cultured in Dulbecco's Modified Eagle's Medium/Nutrient Mixture F12 (DMEM/F12, Gibco) supplemented with 10% FBS and 1% penicillin-streptomycin. In view of the low adherence properties of SSCs, non-adherent SSCs were collected repeatedly and purified. Finally, the culture plates were placed in a 37 °C incubator with 5% CO_2_. SSCs were identified by immunofluorescence staining of ubiquitin carboxyl-terminal esterase L1 (UCHL-1) and glial cell line-derived neurotrophic factor family receptor alpha 1 (GFRα-1). All experiments on animals were approved by the Ethics Committee of Xi'an Honghui Hospital affiliated to Xi'an Jiaotong University.

### Attachment and proliferation of SSCs on various PCL membranes

The attachment of SSCs were detected using the cell counting method. The purified SSCs were seeded on PCL (control group), PCL/Nap-E7 (Nap-E7 group), and PCL/Nap-E7-YIGSR (Nap-E7-YIGSR group) aligned fibrous membranes, which were placed in 24-well plates (1×10^4^ cells per well) of the same diameter. For attachment assays, SSCs were cultured in serum-free DMEM/F12 medium for 2, 4, 6, 12, and 24 h. At each time point, the whole culture medium was gently aspirated and the adhered cells re-suspended. For the proliferation assay, the attached cells numbers were counted with a Cell Counting Kit-8. SSCs were seeded on PCL (control group), PCL/Nap-E7 (Nap-E7 group), and PCL/Nap-E7-YIGSR (Nap-E7-YIGSR group) aligned fibrous membranes, which were placed in 24-well plates (1×10^4^ cells per well) of the same diameter in serum-free medium for 1, 3, 5, and 7 d. The proliferation rate was tested by CCK-8. The proliferation data were normalized to the control group on day 3. Cell adhesion and proliferation rates were calculated. Three replicate wells were set for each group at each time point.

### Cell live/dead assay

The Calcein-AM/propidium Iodide (PI) cell viability assay (Beyotime Biotech, China) was used to evaluate the effect of Nap-E7 or Nap-E7-YIGSR self-assembled peptides on SSCs viability. The staining was performed according to the steps described in the previous article 34, and samples were observed under a fluorescence microscope.

### Neuronal differentiation of SSCs on modified aligned PCL fibrous membranes

The purified SSCs were seeded on the PCL (control group), PCL/Nap-E7 (Nap-E7 group) and PCL/Nap-E7-YIGSR (Nap-E7-YIGSR group) fiber membranes in 24-well plates (1×10^4^ cells per well). The SSCs in three groups were cultured in the DMEM/F12 basal medium, containing 10% FBS, 100 ng/mL fibroblast growth factor (FGF), 10 ng/mL glial cell-derived neurotrophic factor (GDNF), 150 ng/mL sonic hedgehog (SHH), 0.1 mM β-mercaptoethanol (β-ME), 1×10^-4^ mM retinoic acid (RA), 1 mM sodium pyruvate and 1% penicillin-streptomycin. Cells were cultured in an incubator at 37 °C and 5% CO_2_ with medium changes every 2-3 days. Due to the lack of adhesion peptide, the SSCs in the control group were partially washed off during medium changing and staining processes. After 21 days of culture, the neuronal differentiation in each group was identified and analyzed.

### Whole-cell patch clamp electrophysiology

After inducing the differentiation of SSCs into neurons for 3 weeks, the induced neurons were transferred to artificial cerebrospinal fluid (ACSF) and subjected to electrophysiological examination. The patch clamp electrophysiological studies were conducted according to the previously described methodology 36. The whole-cell recording was performed in voltage- or current-clamp mode. The sodium/potassium currents, APs, and spontaneous synaptic currents were measured by Axopatch 200B.

### Immunofluorescence staining assay

The purified SSCs and different PCL membranes were fixed with 4% paraformaldehyde for 15 min and permeabilized with 0.25% Triton X-100 for 10 min. Then samples were blocked with 10% blocking buffer for 1 h and incubated overnight at 4 °C with the following primary antibodies: rabbit anti-UCHL-1 (Abcam, 1:200), mouse anti-GFRα-1 (Abcam, 1:200), mouse anti-Tuj-1 (Abcam, 1:200). After washing with PBS, the samples were incubated in Alexa Fluor 488 or Alexa Fluor 594-conjugated secondary antibodies at 37 °C for 2 h, and DAPI was used to stain the nuclei. Finally, the samples were observed under a laser confocal scanning microscope.

### Western blotting analysis

Western blot analysis was used to detect the expression levels of Tuj-1, BDNF, CGRP, ChAT, integrin β1, GSK3β, p- GSK3β, β-catenin, and cyclin D1 proteins in the cells of three groups. The samples were lysed in RIPA lysis buffer for collecting total proteins. Proteins of each group were separated by SDS-PAGE, transferred to a PVDF membrane, and blocked with 5% skimmed milk powder for 2 h. Primary antibodies, Tuj-1 (Abcam, 1:5000), BDNF (Proteintech, 1:3000), CGRP (Abcam, 1:3000), ChAT (Abcam, 1:5000), integrin β1 (Proteintech, 1:3000), GSK3β (Proteintech, 1:3000), p-GSK3β (CST, 1:5000), β-catenin (Abcam, 1:3000) or cyclin D1 (CST, 1:5000) were added, incubated overnight at 4 °C, and washed in TBST (10 min × 3 times). The membranes were then incubated with the corresponding secondary antibodies (1:1000) at room temperature for 2 h. ECL luminescence reagent was added for color reaction. The ratio of the target protein band's gray value to the gray level of the β-actin band reflected the expression level of the target protein.

### Spinal cord injury model and cell transplantation

SD rats were anesthetized with 1% sodium pentobarbital (50 mg/Kg), and the T8 vertebral plates were removed bilaterally, and the spinal cord was exposed. Briefly, we clamped the spinal cord for 30 seconds using tiny artery clamps with a tip width of 0.5 mm. Subsequently, the wound was cleaned with saline and aseptically sutured. Successful manifestations of the spinal cord injury model included T8 spinal cord hemorrhagic edema, hind limb contraction, caudal spastic swaying, and hind limb sensory deficits. Complete paralysis of both hind limbs was confirmed one day after the model preparation and cells were transplanted into the area of injuried spinal cord. A microinjector was used to pump 10 μL of saline or a suspension containing 1×10^6^ cells into the area of injury at a rate of 1 μL/min. After injecting the cell suspension, the glass pipette tip (80-90 μm diameter) was kept in the spinal cord for an additional 5 minutes to prevent leakage of the cell suspension from the injured area.

### Behavioral tests

#### Gait Analysis

Gait analysis was performed 1, 2, 3, 5, and 7 weeks after transplantation to assess the recovery of motor function. As an automatic quantitative gait analysis system, the Catwalk XT (Noldus) system was used to detect subtle changes in the gait-speed sample rate and reveal neurological gait disorders. The footprints were captured while the rat voluntarily traversed a glass plate towards a goal box. At least three perfect runs were needed for each group per time point for the analysis. Besides, according to the measurement results, the CATWalkXT10.5 system was used to calculate Basso-Beattie-Bresnahan (BBB) score of each rat.

#### Rump-height Index assay

Rats walked across a corridor, over a glass plate. (160 cm long, 10 cm wide, and 8 cm thick) on 1 day prior to and 1, 2, 3, 5, and 7 weeks post SCI. The rump-height Index (RHI) is defined as the height of the rump, normalized to the thickness of the beam measured along the same vertical line 37. To minimize preoperative changes in the RHI of each animal, a standardized RHI (the post-injury value divided by the pre-injury value) was used for comparison.

### Muscle atrophy analysis

The gastrocnemius muscle was dissected out and weighted before fixation. The entire 5 sections of the gastrocnemius muscle of each rat were selected for hematoxylin and eosin (H&E) staining. Images of random fields of each muscle section were captured under a bright-field microscope (Leica DMIRB) at a typical magnification of 200x. The area of the gastrocnemius muscle fibers was analyzed using ImageJ software.

### Spinal Cord Electrophysiology

Spinal cord electrical signal conduction parameters were evaluated using a small animal electrophysiological detector according to the previously described techniques 38. Briefly, a bipolar stimulation electrode consisting of a pair of 85 mm tungsten microelectrodes (0.5 MOhm, KeDouBC.) were placed 1 mm apart and inserted 300-500 μm into the T6 segment of the spinal cord. Single square wave pulses (0.1 ms) were transmitted at 3 second intervals. A 1 mm diameter silver sphere electrode placed at T10 recorded the electrical signal.

### BDA injection

Tract-tracing of axons was performed by BDA (Dextran, biotin, 10000 MW, D2326025, lysine fixable, Invitrogen) injection using a microinjection pump. BDA was prepared at 10% w/v in saline, and 4 × 0.5 μL was injected into bilateral motor cortices, four sites per hemisphere 5 weeks post injury 39. Two weeks later, these rats were sacrificed. BDA labeling was evaluated by Alexa Flour 594 streptavidin (YSASEN, CAT:35107ES60).

### Histological evaluation of recovery after spinal cord injury

Seven weeks after spinal cord injury, rats were deeply anesthetized using 10% chloral hydrate, perfused transcardially with ice-cold heparinized (10 U/mL) saline, and then injected with 4% PFA in PBS. The spinal cord was dissected, and fixed in 4% PFA solution in 0.1 M PBS for 48 h at 4°C. The 8-mm-long spinal cord tissue centered on the lesion was frozen and sliced in 20 μm thick. In brief, the sections were rehydrated in PBS for 5 min followed by incubation with 1% bovine serum albumin and 0.01% triton-X100 for 1 h at room temperature. Then the sections were exposed to appropriate primary monoclonal antibodies (1:300 Tuj-1, Abcam; 1:300 GFAP Abcam; 1:500 MBP; 1:200 Synaptophosin) in a humidified chamber at 4 °C overnight. After washing three times with PBS, the slides were then incubated with an Alexa Fluor 594-conjugated goat anti-mouse secondary antibody (1:800, Molecular Probes) or Alexa Fluor 488-conjugated goat anti-rabbit secondary antibody (1:400, Molecular Probes) at 4 °C in the dark for 2 h. Finally, the sections were mounted onto glass slides for microscopic analysis after staining with 4',6-Diamidino-2-phenylindole (DAPI) and following extensive rinsing with PBS. The cellular and morphological structures of the spinal cord tissue were assessed by H&E staining.

### Statistical analysis

All data were presented as the means ± standard deviation (SD). SPSS 20 (IBM, Armonk, NY, USA) was used for analysis. Differences among the groups were analyzed using one-way variance analysis (ANOVA) followed by a *post-hoc* comparison. All tests were 2-tailed, and *p* < 0.05 was considered statistically significant.

## Results

### Characterizations of aligned PCL fibrous membranes coated with Nap-E7-YIGSR

**Figure 1:**
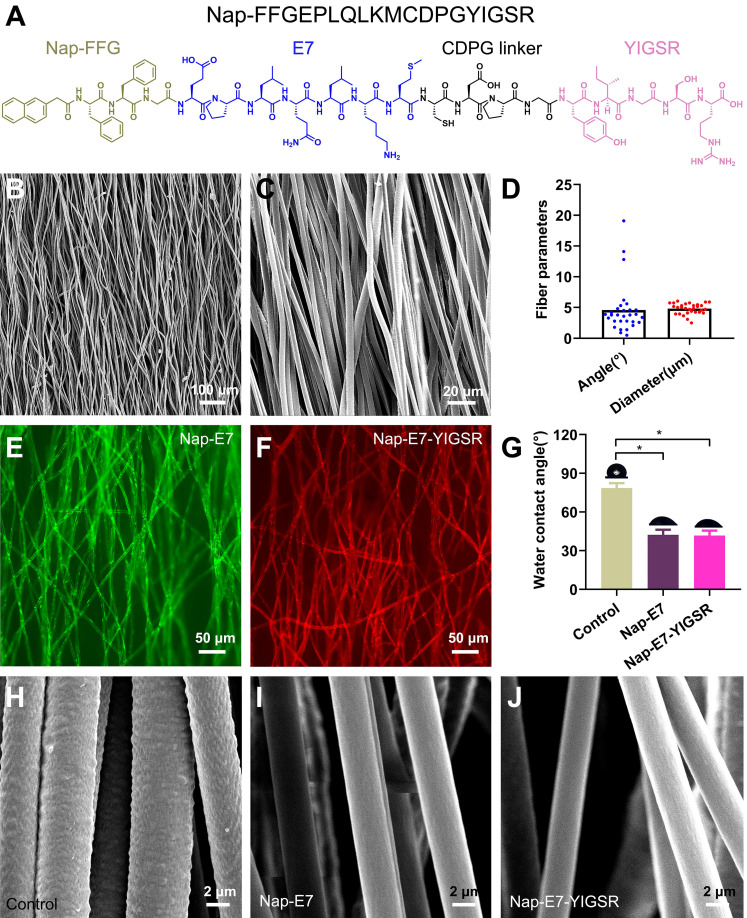
** Structure characterization of aligned PCL fibrous membranes and self-assemble peptides modification. (A)** Chemical structure of Nap-FFGEPLQLKMCDPGYIGSR (Nap-E7-YIGSR) peptide. **(B-C)** Scanning electron microscopy images for the surface of the aligned PCL fibrous membrane under low and high magnification. **(D)** Characteristics of aligned PCL fibrous membrane (*N*=30). **(E-F)** Fluorescence image of aligned PCL fibrous membrane coated with (E) FITC labeled Nap-E7 peptide or (F) rhodamine-labeled Nap-E7-YIGSR. **(G)** Water contact angle measurements (*N*=6, * *p* < 0.05). PCL fibers surface morphology in control **(H)**, Nap-E7 **(I)**, and Nap-E7-YIGSR **(J)** groups.

The Nap-E7-YIGSR and Nap-E7 self-assembled peptides were synthesized by the solid-phase peptide synthesis method. The chemical structure of Nap-E7-YIGSR is displayed in Figure 1A. The Nap-FFGEPLQLKMCDPGYIGSR peptide was composed of a hydrophobic segment (Nap-FFG), stem cell affinity peptide (EPLQLKM), and nerve regeneration-related short peptide sequence (YIGSR). In addition, the aligned PCL fibrous membranes were fabricated by electrospinning, and their representative SEM images are shown in Figure 1B and C. Considering the longitudinal axis of the conduit as a reference direction, the average angle of fibers in the aligned PCL fibrous membrane was 4.5 ± 3.9° (Figure 1D) and an average fiber diameter of 4.8 ± 0.8 μm, indicating that the fibers were of a consistently uniform thickness and tightly organized.

To visualize the distribution of peptide coating within the PCL membrane and evaluate the peptide-binding capacity, Nap-E7-YIGSR and Nap-E7 were fluorescently labeled by fluorescein isothiocyanate (FITC) or rhodamine, respectively. After soaking the functional peptides for 24 hours and rinsing in PBS 3 times, the surface of PCL fibers was evenly and tightly coated with the Nap-E7 (green) and Nap-E7-YIGSR (red) (Figure 1E-F). Due to the strong hydrophobicity of PCL fibers, the hydrophilic angle of the PCL membrane coated with two self-assembled peptides was significantly lower than that of the control group (PCL fiber membrane) (Figure 1G). This result showed that Nap-E7 and Nap-E7-YIGSR modifications could significantly improve the hydrophilicity of the PCL fiber membrane. Besides, since PCL was composed of three elements C, H, O. Nap-E7-YIGSR and Nap-E7 had N elements in addition to C, H, O elements. Based on this, we used X-ray Photoelectron Spectron (XPS) to detect the content of C, N, O elements on the surface of the material to verify the success of the material modification. The XPS results showed that the surface of the modified material contained the element N. And the atomic concentration of element N as a percentage of the total element were 3.2% (PCL+ Nap-E7), 4.1% (PCL+ Nap-E7-YIGSR). Also, SEM images illustrated that the surface of PCL fibers modified by peptides was smoother than unmodified PCL (Figure 1H-J). These results indicated that Nap-E7-YIGSR and Nap-E7 could successfully attach to the surface of PCL fibers and improve their hydrophilicity significantly. The total amount of bound peptides on both membranes (NAP-E7: 0.141 μmol, NAP-E7-YIGSR: 0.143 μmol,) was almost the same after BCA Protein assay.

### Nap-E7-YIGSR-modified aligned PCL fibrous membranes support SSCs proliferation and attachment

**Figure 2:**
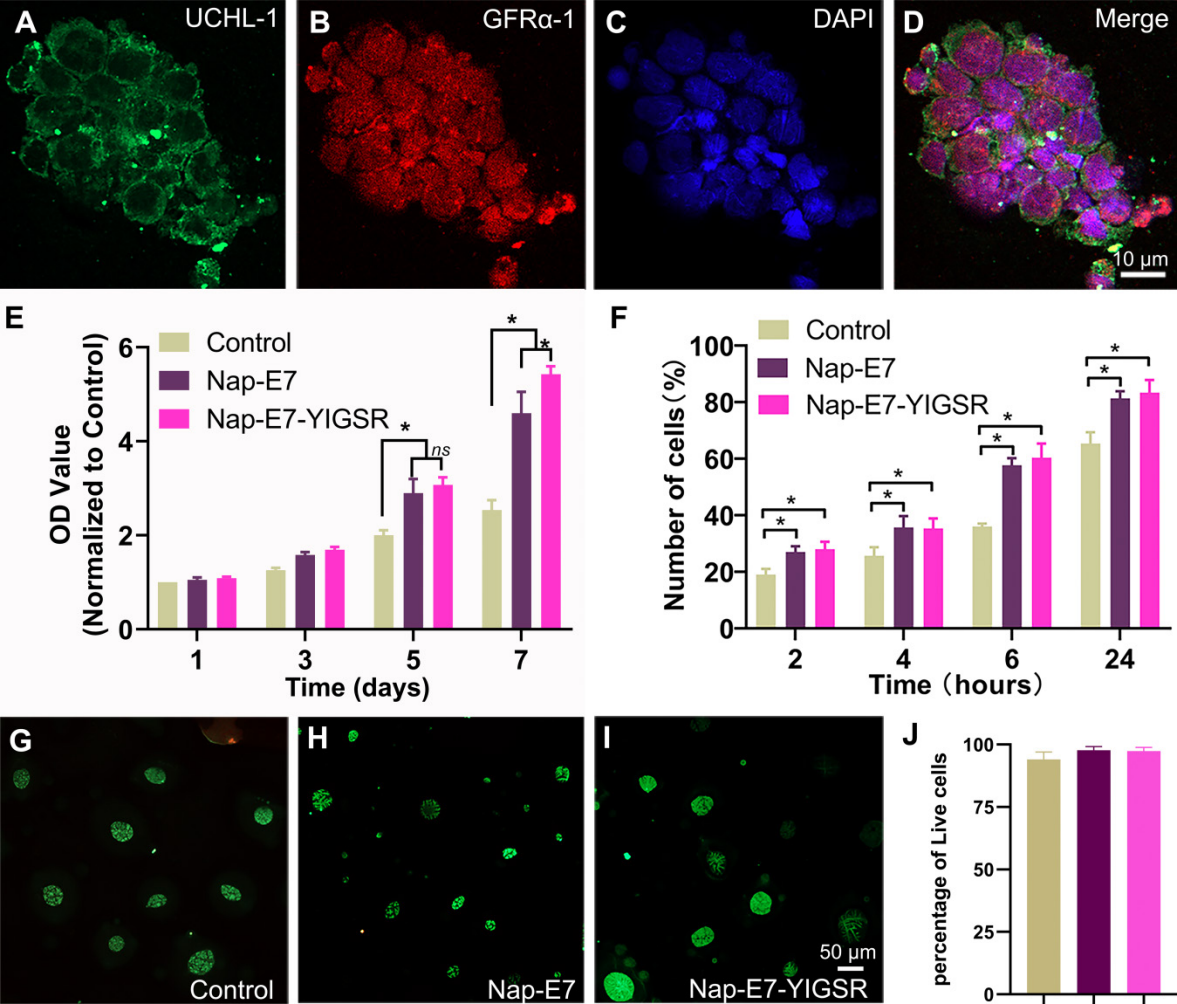
** The identification of SSCs and the cell viability of the SSCs in fibrous film (A-D)** Characterization of SSCs with immunofluorescent staining by the expression of UCHL-1 and GFRα-1. 4′,6-diamidino-2-phenylindole (DAPI) nuclear counterstaining. **(E)** Proliferation assays of the SSCs grown in serum-free DMEM/F12 medium for 1, 3, 5, and 7 d (* *p* < 0.05). **(F)** Attachment assays of the SSCs attached to coated and uncoated films of control, Nap-E7 and Nap-E7-YIGSR aligned fibrous film in serum-free DMEM/F12 medium on 2, 4, 6, and 24 h (* *p* < 0.05). **(G-I)** Fluorescence images of Calcein-AM/propidium Iodide (PI) staining of SSCs grown in three groups. **(J)** Live-dead cell analysis of SSCs using Calcein-AM/PI staining was performed with a fluorescence inversion microscope system 24 h after the SSCs attached to three groups.

Primary SSCs were isolated from the testis tissues of male SD rats aged 23-25 days. Due to the difference in the adhesion characteristics of stromal cells and SSCs, the high purity of SSCs could be obtained through the adherence screening method. The purified SSCs were identified by UCHL-1 (green) and GFRα-1 (red) fluorescence double-label staining (Figure 2A-D). SSCs grew in clusters with a purity of up to 99%.

A Cell Counting Kit-8 (CCK-8) assay was performed to evaluate the proliferation of purified SSCs on modified and unmodified PCL fiber membranes and cultured for 7 days to observe their proliferation (Figure 2E). The proliferation of SSCs on Nap-E7 and Nap-E7-YIGSR coated membranes was significantly higher than that on uncoated PCL membranes, indicating that E7 or E7-YIGSR modification can increase the proliferation of SSCs (p < 0.05). The proliferation of SSCs on Nap-E7 and Nap-E7-YIGSR coated membranes increased by 2.3-fold compared with that on unmodified PCL fiber membranes after 7 d of culture (p < 0.05), indicating that Nap-E7 and Nap-E7-YIGSR modification has a stronger ability in enhancing the proliferation of SSCs than control group. In addition, the Nap-E7-YIGSR group had a similar number of SSCs to the Nap-E7 group on days 3 and 5, but the number was slightly higher on day 7 than the Nap-E7 group (p < 0.05).

A 24 h attachment test was used to test the effect of the E7 and YIGSR on the attachment efficiency of SSCs. Further results suggested that the ratio of attached SSCs on Nap-E7-YIGSR coated membranes (2 h, 28.10%; 4 h, 35.30%; 6 h, 60.35%; 24 h, 83.24%;) or Nap-E7 coated membranes (2 h, 27.01%; 4 h, 35.67%; 6 h, 57.67%; 24 h, 81.33%;) was significantly higher than that of uncoated film in Control group (p < 0.05) (Figure 2F).

The cytotoxicity of Nap-E7 or Nap-E7-YIGSR peptides on SSCs was analyzed by staining SSCs with a live/dead reagent (Calcein-AM/PI). The SSCs in the control group were partially washed off during the medium change and staining process due to the absence of adhesion peptides. The ratio of green-labeled viable SSCs in all three groups was higher than 98% (p > 0.05) (Figure 2G-J). This result indicated that Nap-E7 or Nap-E7-YIGSR peptides were not toxic to SSCs.

### Nap-E7-YIGSR promotes the differentiation of SSCs into neurons

**Figure 3:**
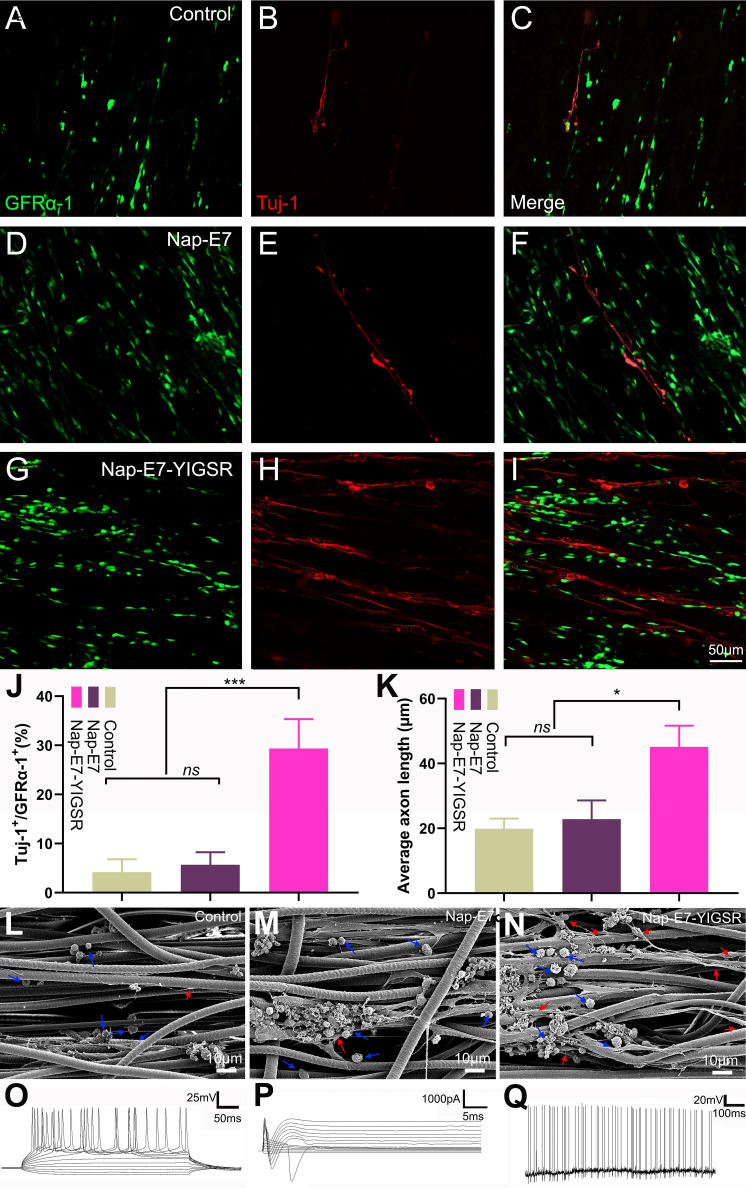
** The differentiation efficiency of SSCs into neurons was enhanced by Nap-E7-YIGSR peptide modification.** Representative immunofluorescence images and quantification of SSCs cultured in PCL film, control group **(A-C)**, Nap-E7 group **(D-F)**, and Nap-E7-YIGSR group **(G-I)** after 21 days through GFRα-1 (SSCs maker, green) and Tuj-1 (neuron marker, red) staining **(J)** (*N*=6, *** *p* < 0.001). The morphology of SSCs and their differentiated neurons on the surface of aligned PCL fibers without coating or coated with Nap-E7 or Nap-E7-YIGSR** (L-N)**. The blue arrows in (L-N) indicate the SSCs and red arrows indicate neurons. **(K)** The average axon length of differentiated neurons derived from SSCs (*N*=6, * *p* < 0.05). Electrophysiological properties of SSC-Derived iNs. **(O)** Representative traces of membrane APs in response to step depolarization via current injection (bottom). The membrane action potential was current-clamped at approximately -65 mV. **(P)** Voltage clamp recording shown putative voltage-gated Na^+^ (inward) and K^+^ (outward) currents. Voltage pulses shown below (-90 to +50 mV). Holding potential was -70 mV.** (Q)** Spontaneous action potentials were recorded from 21-dpi iNs. There was no current injection applied.

The efficacy of Nap-E7 and Nap-E7-YIGSR on neuronal differentiation of SSCs was verified by seeding the SSCs on the surface of aligned PCL fibrous membranes coated with Nap-E7 or Nap-E7-YIGSR and culturing in DMEM basal induction medium. The neuron-specific protein marker of Tuj-1 was detected by immunofluorescence staining to identify the induction efficiency of SSCs to neurons in three groups. The three groups of SSCs showed an orderly growth on aligned PCL fibrous membranes. In the control and Nap-E7 groups, red fluorescence-labeled Tuj-1 positive neurons were sporadically distributed (Figure 3A-C). In addition, the number of green fluorescence-labeled GFRα-1-positive SSCs in the Nap-E7 group was significantly higher than those in the control group (Figure 3D-F). This indicated that the Nap-E7 polypeptide could promote the adhesion of SSCs onto the surface of PCL filaments but could not significantly promote the differentiation of SSCs into neurons compared to the control group. However, the number of neurons produced from SSCs in the Nap-E7-YIGSR group obviously increased compared to the Nap-E7 and control groups. The red fluorescence-labeled neuron axons extended longitudinally along the aligned PCL fibers (Figure 3G-I). A statistical analysis of SSCs differentiation was performed across six randomly selected view-fields of the three groups. The percent of neurons derived from SSCs was approximately 29.3% ± 6.5 (n=6) in the Nap-E7-YIGSR group. In the control and NAP-E7 groups, the percent of neurons derived from SSCs was only 3.3% ± 2.7 (n=6), 5.1% ± 3.1 (n=6). This suggests that the Nap-E7-YIGSR functional polypeptide strongly affected the differentiation efficiency of SSCs to neurons in the DMEM Basal induction medium (Figurer 3J).

To further observe the morphology and axon length of neurons differentiated from SSCs in three groups the samples were fixed, sprayed with gold, and photographed by a scanning electron microscope. As shown in Figure 3L-N, SSCs adhered to fibers in a round or oval shape in three groups, and the SSCs tended to grow in clusters. Besides, the axons of differentiated neurons in all groups extended along the aligned fibers. The average axon length of differentiated neurons in the Nap-E7-YIGSR group was longer than that of the Nap-E7 and control groups (Figure 3K) (Control group: 19.90 ± 3.2 μm, Nap-E7 group: 22.02 ± 5.8 μm, Nap-E7-YIGSR group: 45.10 ± 6.1 μm) (p < 0.05). In addition to enhancing the differentiation of SSCs into neurons, the Nap-E7-YIGSR also promoted the axon growth of differentiated neurons.

To detect the electrical activity in induced neurons, functional patch clamp electrophysiological study was performed on single cells to determine whether the cells derived from SSCs had electrical activity. The membrane action potential characteristics were detected when injecting a series of positive currents to neurons (Figure 3O). In addition, sodium and potassium currents could be elicited from the newly induced neurons (known as sd-iNs) in response to a series of voltage pulses (Figure 3P). Furthermore, spontaneous action potentials were observed in the iNs (Figure 3Q). Collectively, our data indicated that differentiated SSC-derived neurons were mature and functional following the differentiation-inducing process.

### Nap-E7-YIGSR improves neuronal induction and differentiation of SSCs through the integrin β1/GSK3β/β-catenin signaling pathway

**Figure 4:**
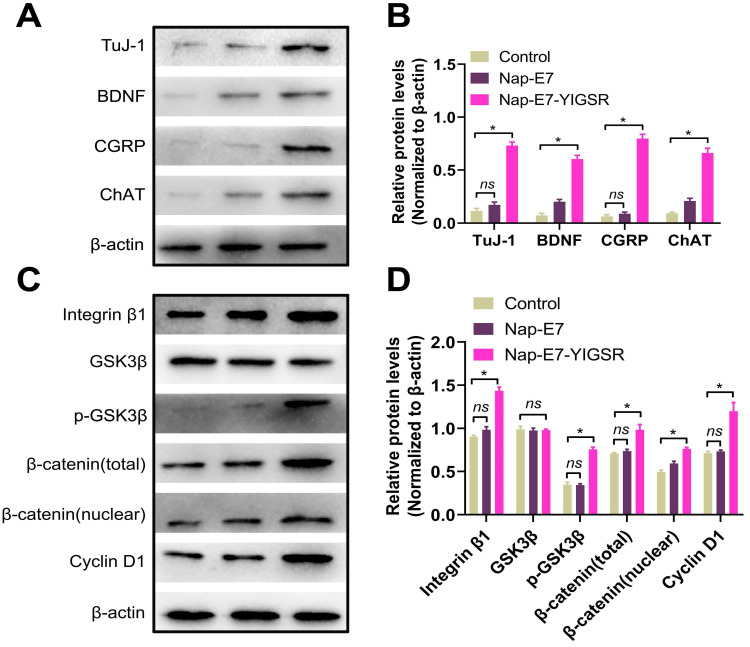
** Western blot analysis of the expression of the characteristic proteins of neurons derived from SSCs. (A)** Western blot bands of Tuj-1, BDNF, CGRP, ChAT, and β-actin in control (Left), Nap-E7 (Middle), and Nap-E7-YIGSR groups (Right). **(B)** Expression levels from three replicates per group were quantified, and average levels are shown relative to β-actin. The integrin β1/GSK3β/β-Catenin signaling pathway was activated in SSCs when cultured on Nap-E7-YIGSR peptide-modified PCL films. **(C)** Western blot bands of integrin β1, GSK3β, p-GSK3β, β-catenin(total), β-catenin(nuclear), cyclin D1, and β-actin in control (Left), Nap-E7 (Middle), and Nap-E7-YIGSR groups (Right). **(D)** Expression levels from three replicates per group were quantified, and average levels are shown relative to β-actin. (*N*=3, * *p* < 0.05).

We next explored the characteristics and function of differentiated neurons and the possible mechanisms underlying the effect of Nap-E7-YIGSR on SSCs neuronal induction. Figure 4A-B showed that the differentiated neurons from SSCs by Nap-E7-YIGSR activation significantly expressed motor neuron marker, choline acetyltransferase (ChAT), sensory neuron marker calcitonin gene-related peptide (CGRP), and brain-derived neurotrophic factor (BDNF) compared to the control group and Nap-E7 group. These data indicated that Nap-E7-YIGSR could induce SSCs to differentiate into different types of neurons. Small-molecule peptides often interact with cell adhesion molecules to perform their biological functions. We explored the mechanism of Nap-E7-YIGSR promoting the induction of SSCs into neurons and found that the expression of integrin β1 increased in the Nap-E7-YIGSR group compared to Nap-E7 and control groups (p < 0.05) (Figure 4C-D). Also, GSK3β was substantially activated in the Nap-E7-YIGSR group by phosphorylation, and the ratio of p-GSK3β/GSK3β was significantly higher than that of other two groups (p < 0.05). In addition, the relative levels of total β-catenin and nuclear β-catenin were increased, and the expression of the transcriptional regulator of cyclin D1 was significantly elevated (p < 0.05) in the Nap-E7-YIGSR group compared with Nap-E7 and control groups. However, there was no statistical difference between Nap-E7 and control groups in p-GSK3β and cyclin D1 expression (p > 0.05). These results indicated that Nap-E7-YIGSR might activate the integrin β1/GSK3β/β-catenin signaling pathway to improve neuronal induction and differentiation of SSCs.

### Transplantation of SSC-derived iNs in rats improves histology and motor function after SCI

**Figure 5:**
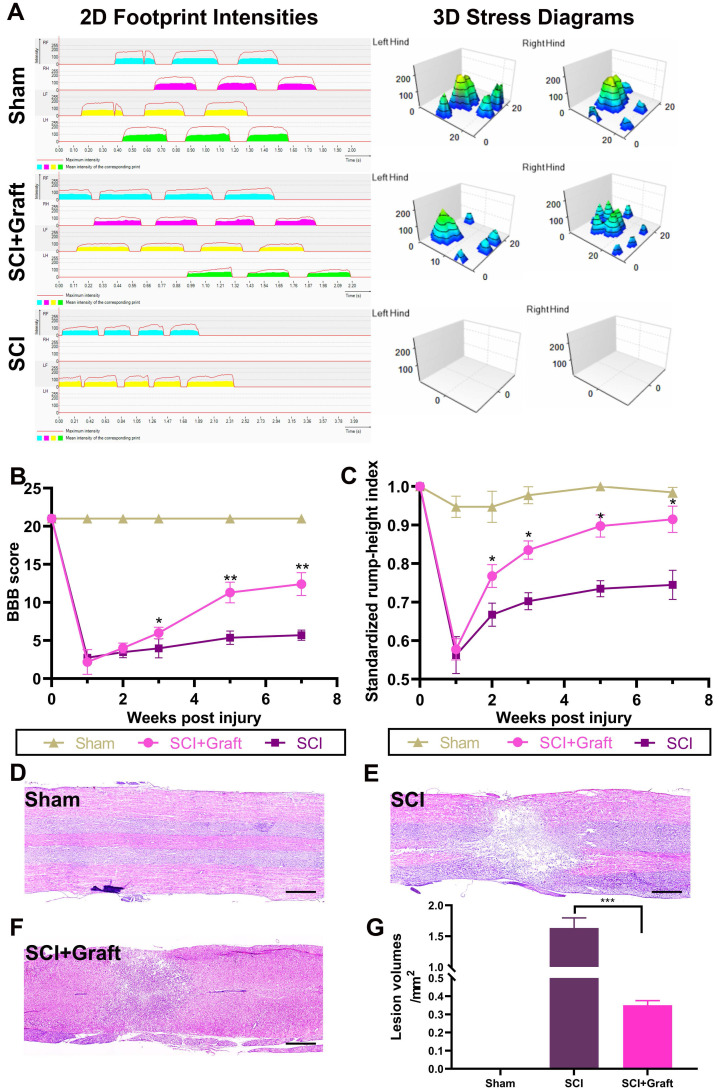
** The results of gait analysis demonstrated motor function recovery. (A)** Representative 2D and 3D stress diagrams at 7 weeks after transplantation are shown. LF = Left front (Yellow), RF = Right front (Blue), LH = Left hind (Green), RH = Right hind (Red). **(B)** BBB scores at 1, 2, 3, 5, and 7 weeks after spinal cord injury. The BBB scores of the grafted group were significantly higher than those of the control group at 1, 2, 3, 5, and 7 weeks after injury. **(C)** Standardized RHI (SRHI) values at 1, 2, 3, 5, and 7 weeks after spinal cord injury. Rump height was significantly higher in grafted rats at 1, 2, 3, 5, and 7 weeks after injury. **(D-F)** H&E staining images of longitudinal sections at 7 weeks post-injury. Scale bars: 500 µm. **(G)** The cavity area was quantified according to the H&E staining. All data were expressed as mean ± SEM. Differences among groups were determined with one-way ANOVA followed by the least significant difference (LSD) test. *n* = 6 in each group, *p < 0.05, **p < 0.01, ***p < 0.001.

Behavioral assessments following the transplantation of SSC-derived neurons are critical for evaluating possible therapies for SCI in pre-clinical trials. To provide a more detailed and quantitative assessment of motor function, we used the Catwalk system, which enables a comprehensive examination of walking motion via 2D and 3D stress diagrams at 7 weeks following transplantation (Figure 5A). The hind limbs of rats in the SCI group were still paralyzed and unable to withstand the stress after 7 weeks. Therefore, the gait analysis only included data from the forefoot. However, rats received neurons transplantation were able to move both hind limbs slowly and in a controlled manner. However, the support strength remained lower than the sham-operated group and contact time was longer. These data could be visualized in the 2D graph. 'The hind toes of neuronal transplanted rats no longer curled, and their contact surface connection was almost similar to that of sham-operated rats in a typical 3D image. However, the firing force of each toe was still lower compared to the sham-operated group. The size and intensity of each footprint suggested a sustainable therapeutic effect on the recovery of walking motion with sufficient weight bearing.

Additionally, we employed BBB and RHI assays to evaluate locomotor functional recovery one day before the injury and 1, 2, 3, 5, and 7 weeks after the spinal cord injury. Between the 2^nd^ and 7^th^ weeks, SCI+graft (iNs) significantly improved the BBB score compared to the SCI group (n = 6 rats/group, *p <0.05, Figure 5B). Similarly, the RHI was significantly increased starting at the 2nd week in the SCI+graft (iNs) group (n = 6 rats/group, *p < 0.05, Figure 5C).

In the SCI+Graft (iNs) group, H&E staining was used to examine the structure and amount of nerve cell infiltration as well as the development of nerve fibers. The average cavity area in the SCI+Graft (iNs) group (0.36 ± 0.03 mm^2^, Figure 5F) was smaller than that in the SCI group (1.61 ± 0.22 mm^2^, Figure 5E). These findings implied that grafting of SSC-derived neurons into the damaged spinal cord could augment tissue regeneration, leading to improved motor coordination and recovery.

### SSC-derived iNs treatment mitigates low skeletal muscle atrophy and increases the gastrocnemius muscle wet weight

**Figure 6:**
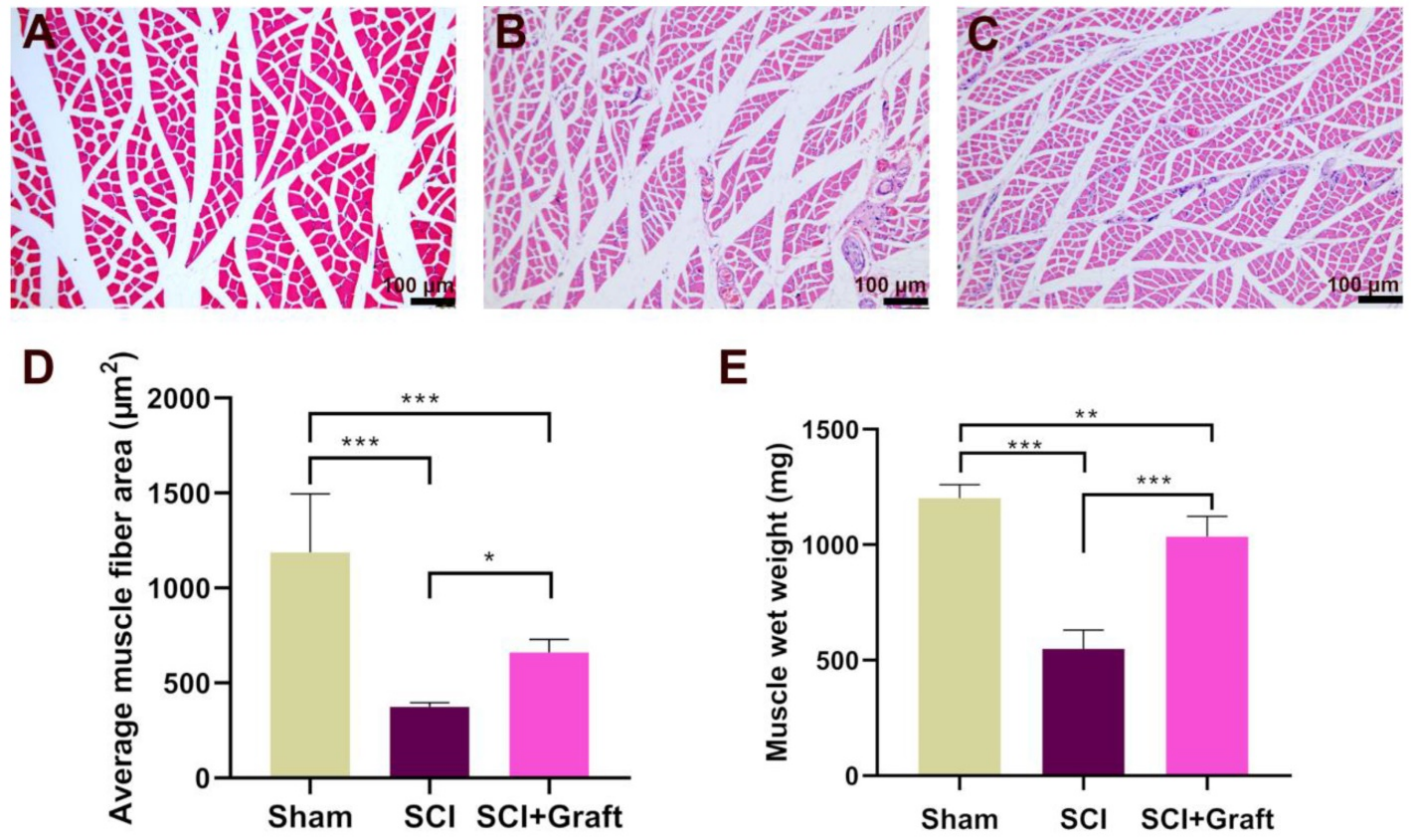
** Skeletal muscle atrophy following spinal cord injury was alleviated by iNs-grafted treatment. (A-C)** Representative muscle fibers stained by hematoxylin and eosin are displayed in the (A) Sham group, (B) SCI group, and (C) SCI+grafted group. **(D)** Quantification of cross-sectional area (µm^2^) of gastrocnemius muscle fibers. **(E)** Showing the change of wet weight of gastrocnemius muscle. Data are presented as mean ± SD. *n* = 6 in each group, *p < 0.05, **p < 0.01, ***p < 0.001, as compared with the SCI group. Scale bars = 100 µm.

Gastrocnemius muscle wet weight and fiber cross-sectional area were analyzed after 7 weeks of SCI to examine the possible development of severe skeletal muscle atrophy. The extent of loss of gastrocnemius muscle was estimated in three groups of rats (Sham group, SCI group, and SCI+Grafted group). SCI produced mild to severe atrophy of the gastrocnemius fibers when compared with Sham group, as shown by H&E staining of transverse gastrocnemius muscle slices (Figure 6A-B). However, the mean muscle fiber area of the SCI+Grafted group was larger than that in the SCI group at 7 weeks (Figure 6C-D). It was demonstrated that the SCI+Grafted treatment could partially ameliorate muscle fiber atrophy. However, the muscle function, as defined by the muscle weight and muscle fiber area, of the SCI+Grafted rats was still not fully recovered compared to the sham group (p < 0.01). Compared to the SCI group, the SCI+Grafted group exhibited significantly more wet gastrocnemius muscle weight at 7 weeks post-injury (Figure 6E, p < 0.001). The iNs-grafted could alleviate muscle atrophy in rats following SCI.

### SSC-derived iNs treatment promotes nerve fiber regeneration and reduces reactive astrogliosis and glial scar formation *in vivo*

Axonal extension through the lesion site is a necessary but not sufficient condition for functional recovery following SCI. We initially determined whether induced neurons (iNs) survived long-term following a 7-week spinal cord injury. The green fluorescence-labeled induced neurons were concentrated in the lesion site. At 7 weeks after transplant, the histological investigation revealed that the transplanted cells had filled the lesion cavity nearly completely. The transplanted neurons extended many axons over great lengths in the rostral and caudal directions. However, no significant axonal elongation was observed in lesion sites without iNs transplantation.

**Figure 7:**
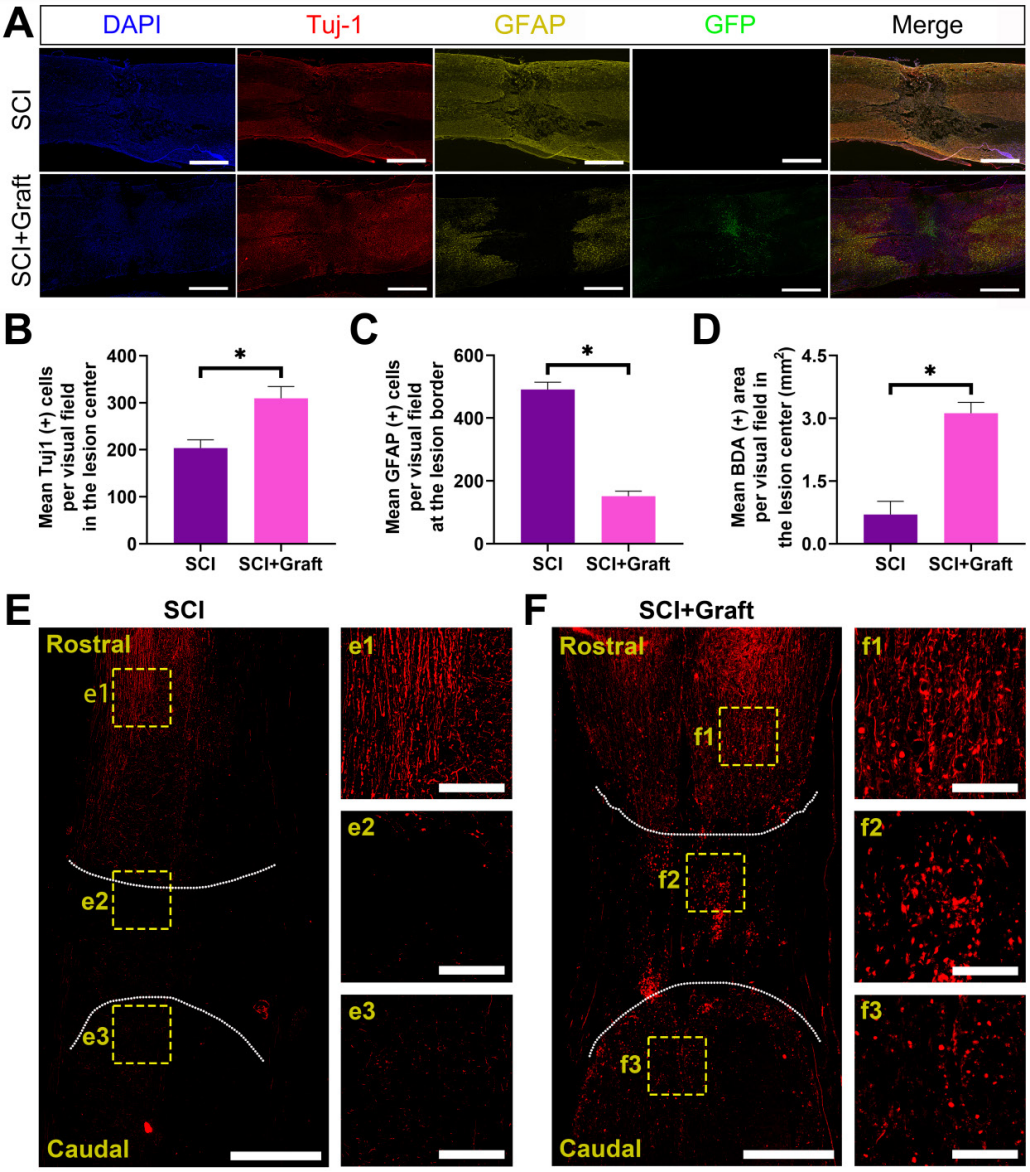
** iNs treatment promoted nerve fiber regeneration and reduced reactive astrogliosis and glial scar formation* in vivo.* (A)** Immunofluorescent staining of Tuj-1 (red) and GFAP (yellow) and GFP (green) at the lesion site. The larger number of GFAP-positive astrocytes was observed in the perilesional area of the injury site in the SCI group, while more Tuj-1-positive cells were found in the SCI+Graft group. Induced neurons (iNs) filled the lesion cavity. **(B-D)** Quantification of immunofluorescence: (B) Mean Tuj-1(+) cells per visual in the lesion center. (C) Mean GFAP (+) cells per visual in the lesion border. (D) Mean BDA (+) cells per visual in the lesion center (mm^2^). **(E, F)** Representative images of BDA-labeled axons in SCI (E), SCI+Grafted (F) groups at day 14 post BDA injection. Scale bars: 1 mm. f1-fb, e1-e3 are the enlarged images of cranial, lesion center, and caudal test, respectively. Scale bars: 250 µm. n=3, * p < 0.05.

Astrocyte proliferation at the lesion site was assessed by GFAP immunostaining, followed by calculating the mean GFAP positive cells per visual around the proximal lesion border. Seven weeks after injury, GFAP was more intensely stained on the rostral and caudal sides of the lesion center in the SCI group, which is indicative of astrocyte proliferation. Seven weeks after the damage, the mean GFAP positive cells per visual were considerably lower in the transplantation group than in the SCI group, suggesting that iNs grafts could help minimize astrocyte proliferation around the lesion (Figure 7A).

Furthermore, the levels of Tuj1 and GFAP were quantified in terms of the mean positive cells per visual field in the lesion center or at the lesion border (Figure 7B-C). For quantification, the Tuj-1 level dramatically increased in the lesion center in SCI+Graft group (301 ± 23 per visual field) compared with SCI group (194 ± 17 per visual field) (Figure 7B). However, GFAP level dramatically increased in the lesion border in SCI group (483 ± 25 per visual field) compared with SCI+Graft group (146 ± 15per visual field) (Figure 7C). Together, these findings indicate that SSC-derived iNs treatment promotes nerve fiber regeneration and reduces reactive astrogliosis and glial scar formation *in vivo*.

In this experiment, the BDA signal was marked in the cerebral cortex, the marking of the SCI site and the nerve fibers near the tail was detected, and the integrity of the nerve fibers in the central nervous system was observed. If BDA could pass through the damaged spinal nerve fibers and establish a signal connection between the two ends of the nerve fibers, it means that the neural circuit in the damaged area has been re-established. There was almost no BDA positive staining on the caudal side of the SCI site in the SCI group after 7 weeks, while there was an apparent BDA positive staining signal on the caudal side of the SCI site in the SCI+Grafted group (Figure 7D-F).

### Axonal remyelination, synapse formation in lesion areas, and electrophysiological improvement by SSC-derived iNs transplantation following SCI

**Figure 8:**
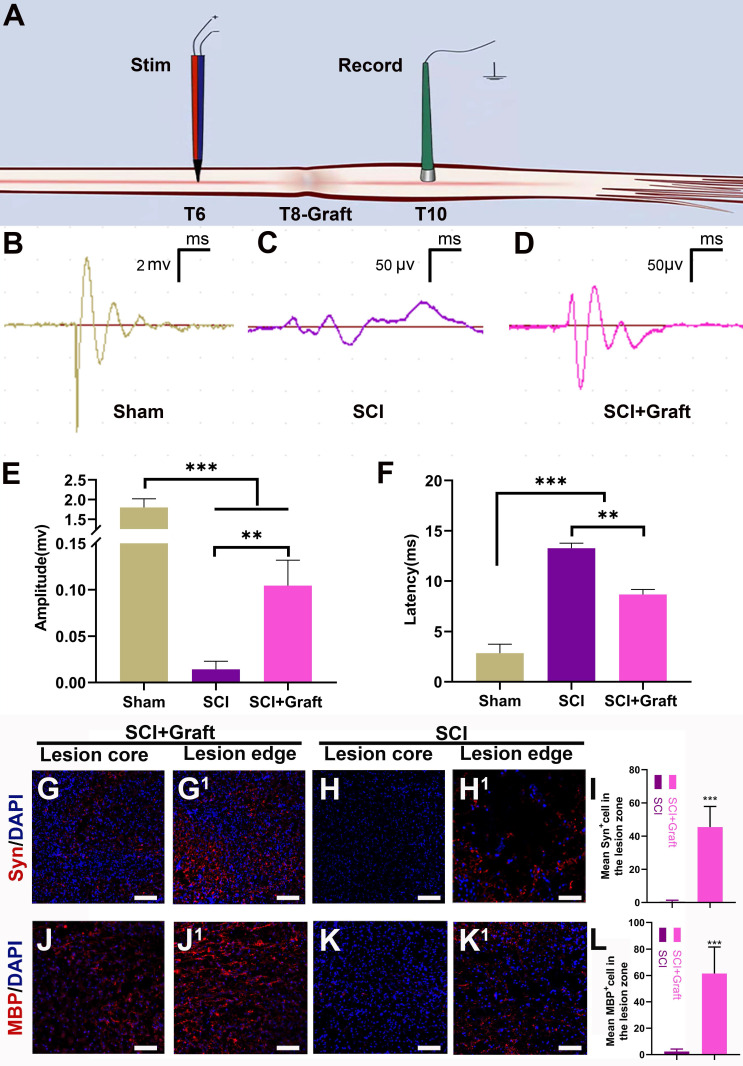
** SSCs derived iNs transplantation induces electrophysiological recovery, axonal remyelination, and synapse formation. (A)** Pattern diagram of stimulation and recording. **(B-D)** Representative images of stimulating potentials in normal rats and rats with different treatments at 7 weeks. **(E-F)** Quantification of amplitude (E) and latent periods (F) in rats of each group at 7 weeks. **(G-L)** Representative immunofluorescence images stained with MBP (myelin basic protein) and Syn (synaptophosin) and quantification showed a wealth of remyelinated fibers and synapses presented in the lesion site of rats in SCI+Graft group and SCI group. Scale bars: 100 µm. Error bars indicate the SD from three different experiments. ** p < 0.01, *** *p* < 0.001. determined by one-way ANOVA.

Electrophysiological tests were performed in three groups to confirm the relationship between functional recovery and axonal regrowth at the site of the damage. A stimulating electrode was placed in the dorsal T6 spinal cord (two spinal segments above the lesion), and recordings were made at T10 (two spinal segments below the lesion) (Figure 8A). In the sham group, stimulation at T6 evoked a short latency (2.84 ± 0.32 ms) response (Figure 8F) that was much shorter than the SCI group (13.26 ± 0.21ms). Longer response latencies were seen in the grafted group (8.67 ± 0.24 ms) when compared to the sham group (2.84 ± 0.32 ms) (p < 0.01), indicating the presence of polysynaptic relays formed by the graft. Response amplitudes in the grafted group (0.11 ± 0.03mV) were smaller than in the sham group (1.80 ± 0.25mV). However, in the grafted group, amplitudes were all moderately restored compared with the SCI group. (Figure 8E) This suggested that the SSC-derived iNs might serve as a “relay” to aid in the reconstruction of damaged neural circuits and the transmission of descending impulses across the SCI/graft site.

To identify the remyelination of regenerated axons, we stained the myelin basic protein marker (MBP) in both SCI+Graft group and SCI group. The results showed many regenerated axons in the lesion zone of rats of SCI+Graft group were successfully remyelinated (Figure 8J- J^1^). MBP^+^ cells staining was rarely observed in the lesion edge or core in the SCI group (Figure 8K- K^1^) (mean MBP^+^ cell number: SCI+Graft group: 60 ± 18; SCI group: 3 ± 1). The formation of synapses was assessed by immunostaining for synaptophysin (Syn). As shown in Figure 8G, G^1^, a dense network of Syn positive signals was detected across the lesion region of rats in the SCI+Graft group. In contrast, Syn staining was rarely observed in the lesion edge or core in the SCI group (Figure 8H-H^1^). These results suggested that SSC-derived neurons could grow axons and form new synapses with proximal and distal axons in the lesion site, and that these nascent axons were wrapped by nascent myelin within the injury microenvironment, which facilitated the precise and rapid transmission of electrical signals.

## Discussion

In this study, we designed and synthesized a new self-assembled peptide Nap-FFGEPLQLKMCDPGYIGSR (Nap-E7-YIGSR), comprising a Nap-FFG hydrophobic terminal that has an adequate capacity to adhere to PCL fibers' surface. Coating with Nap-E7-YIGSR significantly improved the hydrophilicity of PCL electrospinning membranes, by enhancing SSC adhesion and proliferation. Cytotoxicity tests also showed that Nap-E7 or Nap-E7-YIGSR rarely induced necrotic or apoptotic death of SSCs. Additionally, *in vitro* experiments revealed that Nap-E7-YIGSR could significantly promote the induction of SSCs into different types of neurons and promote the growth of differentiated neuronal axons. Furthermore, the Nap-E7-YIGSR enhanced the differentiation of SSCs into neurons by activating the integrin β1/GSK3β/β-Catenin signaling pathway (Figure 4). This directional differentiation protocol for converting SSCs to neurons avoided the drawbacks of traditional induction methods, such as inefficiency, tedious multistep processes, and the high risk of exogenous gene delivery. The therapeutic potential of the induced cells from SSCs was validated in our *in vivo* experiments by behavioral and electrophysiology improvements in SCI rats after iNs transplantation (Figure 8). Innovative SCI clinical therapy will need to focus on safety, efficacy, and practicality.

SSCs are a particular type of stem cells in the testis seminiferous epithelium. Mature SSCs can maintain the stem cell pool through self-renewal and continuously differentiate into spermatocytes and sperms 40. Previous studies have shown that SSCs have pluripotent properties of differentiating into a variety of cell types 41-43. In addition, SSCs exhibit properties similar to ESCs, which can directly differentiate into other cells and tissues in the presence of an adequate inductive milieu. Unlike other adult stem cells, SSCs maintain their activity as they age 20. Therefore, spermatogonial stem cells will have broad application prospects in the field of regenerative medicine. It was reported that mouse SSCs had vascular differentiation abilities *in vitro* 44. Other study confirmed that SSCs could differentiate into renal parenchyma cells *in vivo* 45. The chicken SSCs were reported to differentiate into neuron-like cells, osteoblasts, and adipocytes in different culture medium *in vitro* 42. Studies have also confirmed that SSCs could differentiate into functional neurons and glial cells 21. Thus, these findings further confirmed that SSCs could differentiate into multiple types of cells of all three germ layers and share many features with ESCs. Pig SSCs could be induced into neuron-like cells in the medium containing β-Mercaptoethanol (β-ME), retinoic acid (RA) and 3-isobutyl-1-methylxanthine (IBMX) 43. Another study established that human SSCs could directly differentiate into functional dopaminergic neurons in two phases using RA and multiple growth factors 46. In addition, the neuronal cell types were generated by medaka fish spermatogonial cell line when transfected with mammalian achaete-scute homolog 1 (mash1) 47. Mash1 is a basic helix-loop-helix transcription factor that plays an essential role in driving the differentiation of murine ESCs into neuronal lineages and regulating murine embryonic neurogenesis 48. These results demonstrated that SSCs of different species have many qualities of ESCs that can develop into neuron-like cells under specific culture conditions.

Because neurons in the CNS are incapable of regeneration, neuronal replacement therapy has emerged as the most promising treatment option for CNS degeneration or damage. Neuronal cells differentiated from SSCs provide a novel seed cell for cell transplantation to treat brain and spinal cord injury. However, the SSCs comprise only 0.02-0.03% of the total testis germ cell suspension, and the efficiency of SSC differentiation into neurons *in vitro* is relatively low. Therefore, the most pressing issue in neuronal replacement therapy is to increase the efficiency of neuronal differentiation of SSCs.

SSCs have a low capability for adhesion and frequently suspend in growth media during flask shaking or medium stirring. However, neuronal cells grow adherently *in vitro*. Therefore, promoting the SSC adhesion properties to the culture substrates is the first step to enhance their neuronal differentiation. Polylysine and gelatin are commonly used as coating materials to promote cell adhesion. The RGD peptide sequence is composed of arginine, glycine and aspartic acid. It exists in various types of extracellular matrix and can specifically bind to 11 types of integrins. The fibroblasts, Schwann cells, neurons and other cells are prone to adhere to the biological materials with RGD sequence 49. The short peptide, RNIPPFEGCIWN derived from the LG3 domain of the human laminin α2 chain, displays multiple cellular activities, including cell adhesion, spreading, and migration 50, and the ECM plays an essential role in cell migration, adhesion, and growth.

Additionally, functional peptide motifs in the protein network of ECM have also been discovered. The E7 peptide exhibits a strong affinity for mesenchymal BMSCs. However, little is known about its adhesive ability for SSCs. A previous study found that Nap-FFG has strong hydrophobicity and could bind to water-repellent materials such as PCL filaments. PCL is a bio-inert and non-toxic polymer with a long degradation time of over 24 months *in vivo* and *in vitro*. It can be fabricated into a scaffold of various shapes and is usually suitable for the surface modification of various biological proteins or peptides.

In this study, we covalently linked Nap-FFG and E7 peptides to form a new self-assembled peptide. We found that Nap-FFG-E7 was evenly coated on the surface of PCL filaments. That greatly improved the hydrophilicity of PCL fibers and significantly promoted the adhesion rate of SSCs to the surface of PCL fibers. This indicated that there might be similar polypeptide binding domains on the surfaces of SSCs and BMSCs. YIGSR is a polypeptide sequence derived from laminin that can promote the growth of neuronal axons. We attached YIGSR to the end of the Nap-FFG-E7 polypeptide via a non-functional CDPG linker sequence to increase the efficiency of SSC differentiation into neuronal cells. We found that Nap-E7-YIGSR could enhance the differentiation efficiency of SSCs to neurons compared to Nap-E7 and boost axon growth when cultured in the basal inductive medium. This biological function of Nap-E7-YIGSR is probably due to promote SSCs adhesion on aligned fibers, providing a 3D microenvironment for cell differentiation and axon growth, and YIGSR direct contact to the cell membrane and enhance the sensitivity of SSCs in the basal induction medium. This functional peptide we designed combines self-assembly, adhesion-promoting, and neuronal differentiation functional sequences. In the context of Nap-FFG-E7-YIGSR, the Nap-FFG may self-assemble selectively at the surface of hydrophobic materials without impairing the biological functions of E7 and YIGSR.

Although Nap-E7-YIGSR was found on enhancing SSCs neuronal induction, the possible underlying mechanisms remained unclear. Polypeptides often combine with adhesion molecules, receptors, integrins, and other cell surface molecules to exert biological effects. In our study, western blot analysis revealed that Nap-E7-YIGSR might activate the integrin β1/GSK3β/β-catenin signaling pathway to augment the differentiation of SSCs into neurons. Laminin was shown to bind to β1 integrins and act in association with the actin-based cytoskeleton to attenuate adenylate cyclase activity during cardiac disease or development 51. Moreover, we also confirmed that YIGSR, a peptide sequence derived from laminin protein, could increase the expression level of integrin β1 in SSCs. The GSK3β/β-catenin pathway is required for cell proliferation, differentiation, migration, and nervous system development, which has been proven to regulate neuronal progenitor proliferation, mediate neuronal differentiation of the human cord blood stem cells, and promote BMSCs osteogenic differentiation 52, 53. Our findings revealed that the GSK3β/β-catenin pathway was also involved in the differentiation process of SSCs. However, different types of neurons may exert different therapeutic activities in neuronal replacement therapy regime. Therefore, the related molecular mechanisms of SSC differentiation into different neurons need to be addressed.

We subsequently evaluated the effect of *in vivo* transplantation of SSC-derived iNs on repairing SCI. Although intrinsic neural stem cells of the spinal cord are capable of differentiating into neurons following injury, the number of such neural stem cells is low and functional restoration can hardly be achieved. Exogenous stem cell-derived neurons are essential as transplanting seed cells. Neurons and stem cells face the microenvironment of spinal cord injury after transplantation, and their survival is a prerequisite for subsequent function 5. After 7 weeks of transplantation, the induced neurons in the damaged area were still alive and well, according to our findings. This also confirmed the ability of SSC-derived neurons transplanted in the spinal cord to adapt to the inhibitory and inflammatory environment in the early and middle stages of SCI. It has been demonstrated that MSC-derived neuron-like cells survive for 7 weeks after transplantation into the SCI area 54. Furthermore, studies have shown that neural stem cells produced from induced pluripotent stem cells survive long after being transplanted into injured areas of the spinal cord 55. According to these findings, stem cell-derived neural-like cells allografts do not trigger substantial immune rejection and can efficiently survive in the injured environment. To further verify the recovery of motor function after transplantation, we found that transplantation of SSC-derived neurons could effectively restore motor function in the hind limbs of rats by behavioral evaluation indexes such as BBB scores. After remyelination of regenerated axons and formation of synapses in the lesion site, SSC-derived iNs might eventually rebuild neuronal connection and aid functional recovery.

That's a favorable sign that the transplanted neurons could create new synapses with the spinal cord's intrinsic axons, allowing the proximal nerve electrical signals to reach the distal end successfully. The SCI+iNS group had successful exogenous electrical stimulation transmitted to the distal end of the injured site, whereas in the SCI group there was no such electrical conduction activity across the affected area. Additionally, after SCI, glial scarring and cavitation play a crucial role in neurological function recovery. We found that the cavity volume and glial scar proliferation were smaller in the SCI+ iNs group than that in the SCI group. The extension of spinal nerve axons requires the help of a beneficial ECM substrate, and the formation of cavities prevents the axons from adherent growth and traverse 56. Of course, glial scar, a substrate for axon extension that can be both advantageous and harmful, is not conducive to nerve regeneration through excessive reactive hyperplasia 57. We also revealed that SSC-derived iNs effectively reduced cavity formation and reactive proliferation of glial scars, which might be related to their filling effect and early remodeling of damaged neuronal circuits, indirectly affecting the microenvironment and glial cell state. Liu et al provided evidence that neural stem cell transplantation effectively ameliorated reactive glial cell proliferation and reduced glial scar development after SCI 39, 58. The fact that muscle atrophy has improved indicates that early neural loop remodeling has occurred. Our results showed that SCI+ iNs rats had considerably better ratios of gastrocnemius muscle fiber area in the hind legs than the SCI group. SSC-derived neurons can grow axons to help rebuild damaged nerve pathways and improve gastrocnemius atrophy through reinnervation of lower limb nerves.

## Conclusion

In summary, we have successfully designed and synthesized a functional self-assembled peptide Nap-E7-YIGSR, which exhibited specific characteristics of combining self-assembly on the surface of PCL fibers and enhanced SSC adhesion. In addition, the Nap-E7-YIGSR peptide is capable of effectively differentiating SSCs into multiple types of neurons by regulating the integrin β1/GSK3β/β-Catenin signaling pathway. This novel multifunctional peptide could enhance the neuronal differentiation efficiency of SSCs by imparting instructive physical and biological cues. The mature neurons differentiated from SSCs *in vitro* were functional and could effectively reconstruct neural circuits, participate in the transmission of neural electrical signals, and facilitate the recovery of motor function. We envision that this multi-domain self-assembling peptide may serve as a powerful strategy for neuronal differentiation of stem cells and facilitate neuronal replacement in CNS regenerative medicine.
